# Acidic Sophorolipid Biosurfactant Protects Serum Albumin Against Thermal Denaturation

**DOI:** 10.3390/ijms26178752

**Published:** 2025-09-08

**Authors:** Julia Ortiz, Paulo Ricardo Franco Marcelino, José A. Teruel, Francisco J. Aranda, Antonio Ortiz

**Affiliations:** 1 Departamento de Bioquímica y Biología Molecular-A, Facultad de Veterinaria, Universidad de Murcia, 30100 Murcia, Spain; julia.ortiz@um.es (J.O.); teruel@um.es (J.A.T.); fjam@um.es (F.J.A.); 2 Laboratório de Bioprocessos e Produtos Sustentáveis (LBios), Escola de Engenharia de Lorena (EEL), Universidade de São Paulo (USP), São Paulo 05508-220, Brazil; paulo.franco@ufabc.edu.br

**Keywords:** biosurfactant, sophorolipid, Bovine Serum Albumin (BSA)

## Abstract

Sophorolipids (SLs) constitute a group of unique biosurfactants in light of their unique properties, among which their physicochemical characteristics and antimicrobial activity stand out. SLs can exist mainly in acidic and lactonic forms, both of which display inhibitory activity. This study explores the interaction of non-acetylated acidic SL with bovine serum albumin (BSA). SL significantly enhances BSA’s thermal stability, increasing its midpoint unfolding temperature from 61.9 °C to approximately 76.0 °C and ΔH from 727 to 1054 kJ mol^−1^ at high concentrations, indicating cooperative binding. Fourier-Transform Infrared Spectroscopy (FTIR) analysis confirms SL’s protective effect against thermal unfolding, enabling BSA to maintain its helical structure at 70 °C, distinguishing it from other surfactants that cause denaturation. Furthermore, SL fundamentally alters the sequence of thermal unfolding events; β-aggregation precedes helical domain unfolding, suggesting protective binding to BSA’s helical regions. Computational docking reveals high-affinity binding (Kd = 14.5 μM). Uniquely, SL binds between BSA domains IB and IIIA, establishing hydrophobic interactions, salt bridges, and hydrogen bonds, thus stabilizing the protein’s 3D structure. This distinct binding site is attributed to SL’s amphipathic character. This work deepens the understanding of the molecular characteristics of SL–protein interactions and contributes to improving the general knowledge of this outstanding biosurfactant.

## 1. Introduction

Biosurfactants, which are primary or secondary metabolites produced by animals, plants, or microorganisms, possess an amphipathic structure. This gives them the dual potential as surfactants and emulsifiers, making them a more ecofriendly option than synthetic surfactants [1], alongside displaying distinct biological activities [2]. Over recent years, there has been intensive study and application of microbial biosurfactants, particularly glycolipids like rhamnolipids and sophorolipids (SLs). These compounds are now key components in formulations for agricultural, food, cleaning, cosmetic, and pharmaceutical products [3,4,5].

SLs are glycolipids mainly produced by some yeast of *Candida* and *Starmerella* genera which, among the glycolipidic biosurfactants, have the greatest potential for industrial applications [6]. SLs have garnered significant attention due to their surface-active properties, low toxicity, and biodegradability [7]. These amphiphilic molecules are characterized by a hydrophilic sophorose sugar unit and a hydrophobic fatty acid tail [7]. The unique interplay between their complex structural diversity and their versatile self-assembly behaviors under varying environmental conditions dictates their broad spectrum of applications, ranging from household cleaning to advanced biomedical and nanotechnological fields [8].

**Figure 1:**
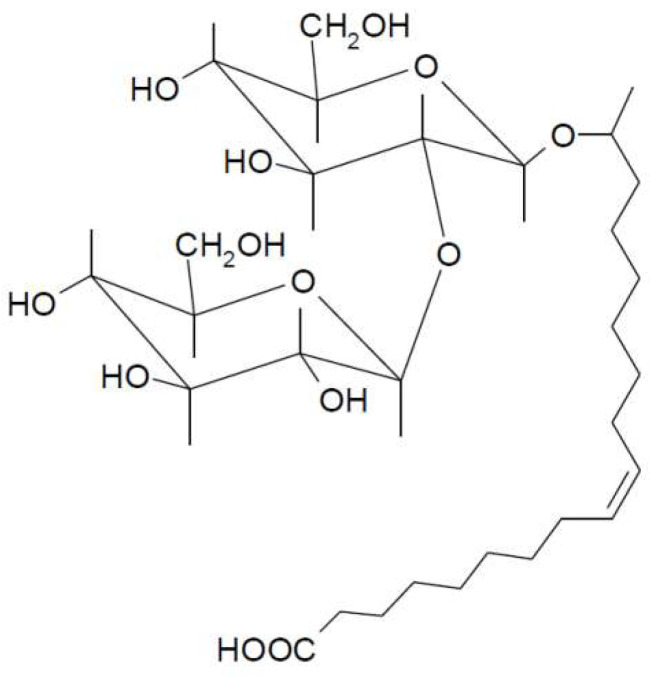
The structure of the non-acetylated acidic SL used in this work: 17-L-[(2-O-D-glucopyranosyl-D-glucopyranosyl)oxy]-*cis*-9-octadecenoic acid.

The fundamental structure of a SL comprises a polar carbohydrate head group, specifically a sophorose unit, which is a glucose disaccharide linked via a β-1,2 bond, and a nonpolar fatty acid tail [7]. The sophorose unit is covalently attached to the fatty acid chain through its 1′ hydroxyl group, typically at the penultimate (ω-1) or terminal (ω) carbon atom of the fatty acid chain [9]. The fatty acid tails commonly range from 16 to 18 carbon atoms in length, although variations can occur depending on the carbon substrates used during production [7]. SLs exist predominantly in two forms: acidic (open-ring) (Figure 1), and lactonic (closed-ring). In the acidic form, the carboxylic end of the fatty acid tail is free. In contrast, the lactonic form has its carboxylic end internally esterified. The ratio of these two forms is influenced by culture conditions and fermentation time [8,9]. Beyond length (C_16_ or C_18_), the fatty acid tail can vary in its degree of unsaturation (saturated, monounsaturated, or polyunsaturated) and the position of hydroxylation (ω-1 or ω) [9,10]. SLs are highly effective biosurfactants due to their ability to significantly lower both surface tension (at the air–water interface) and interfacial tension (at oil–water interfaces). This reduction occurs effectively at extremely low concentrations up to their critical micelle concentration (CMC), which is between 20 and 40 mg L^−1^ [9]. SLs are characterized as relatively hydrophobic compared to some other biosurfactants like rhamnolipids [11]. This higher hydrophobicity aids their role in applications like microemulsion formation and oil removal [11,12].

The remarkable ability of SLs to spontaneously self-assemble into diverse macromolecular structures in aqueous solutions above their CMC is central to their functionality and applications [9,13,14]. The tailored structural features and dynamic self-assembly capabilities of SLs enable their wide-ranging applications. Their surface activity (reducing surface and interfacial tension) and foam-forming abilities (especially acidic forms) make them valuable in household detergents and cleaning agents [7,9,15]. The ability to form microemulsions for detergency is also noted [11,12]. The capacity to form diverse self-assembled structures, such as micelles, vesicles, and nanostructured lipid carriers, positions SLs as promising vehicles for drug delivery [12]. They can encapsulate hydrophobic drugs like curcumin, enhancing its bioavailability [8].

SLs exhibit selective anticancer activity by inducing apoptosis in cancerous cells, often showing low cytotoxicity to normal cells [10,16]. The effectiveness can vary with structural modifications like acetylation and fatty acid composition. The enhanced cytotoxicity on cancer cells is supported by optimal charge and pH interactions between sophorolipid assemblies and cell membranes [16]. Despite their significant advantages, challenges such as higher production costs and variability in properties of crude mixtures compared to uniform synthetic surfactants still exist. However, ongoing research aims to address these limitations through optimized production strategies and genetic engineering to achieve tailor-made SLs for specific industrial and biomedical needs.

Within these wide fields of SL applications, understanding their interactions with proteins constitutes a crucial step for various biotechnological applications. Biosurfactants and proteins can work together to create effective drug delivery systems. By forming structures such as emulsions, hydrogels, and nanoparticles, these systems improve how well drugs dissolve, remain stable, and are released exactly where they are needed. This synergy between biosurfactants and proteins allows for the creation of complex structures that can enhance drug absorption and improve the effectiveness of orally administered drugs. Additionally, these delivery systems may even possess their own antimicrobial or anticancer properties [17,18]. The specific nature and outcome of the SL–protein interaction will depend heavily on factors such as the concentration and type of SL (e.g., lactonic vs. acidic), the protein’s intrinsic properties (e.g., charge, hydrophobicity, secondary structure), pH, and ionic strength of the environment. This paper contributes to elucidating the intricate mechanisms governing these interactions, using bovine serum albumin (BSA) as a model, paving the way for novel applications of SL biosurfactants.

## 2. Results and Discussion

In this work we have used a non-acetylated acidic SL bearing an oleic acid fatty acid chain (Figure 1), on the basis that the number of studies devoted to the study of the physicochemical properties of these acidic SLs and its interactions with proteins are scarce. Non-acetylated acidic SL is a disaccharide consisting of β-1,2′-linked glucose residues with an unsaturated (*cis*-9) 18 carbon fatty acid chain.

### 2.1. Effect of SL on BSA Thermal Unfolding

**Figure 2:**
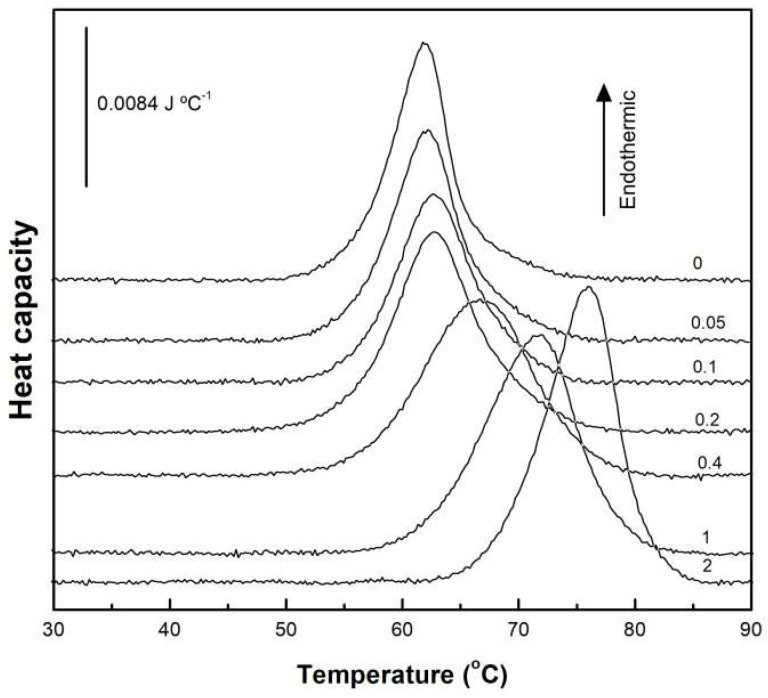
High-sensitivity DSC thermograms for the thermal unfolding of BSA in the absence and presence of increasing concentrations of acidic SL. BSA concentration was 0.1 mM. Numbers on the scans give the mM concentration of SL.

To keep protein aggregation to a minimum, we worked with dilute BSA solutions. Figure 2 presents the DSC thermograms for native BSA, both alone and with increasing concentrations of acidic SL (0–2 mM). Native BSA exhibited an endothermic thermal unfolding process, characterized by a midpoint temperature of 61.9 °C and an average unfolding ΔH of 727 kJ mol^−1^. We confirmed the irreversibility of these transitions through successive scans after cooling the samples. These results were consistent with prior research [19,20,21], with small differences due to different experimental conditions, such as buffer composition, protein concentration, or scan rate. At concentrations of SL equal or below 0.2 mM (SL/BSA molar ratio = 2), the thermal unfolding of BSA was minimally affected. However, an abrupt change was observed above this SL/BSA molar ratio, affecting both Tm and ΔH. At SL/BSA molar ratio of 4, the thermal unfolding transition was considerably widened, probably corresponding to the sum of two superimposed transitions: pure BSA and BSA with bound SL. In fact, further increasing the SL concentration resulted in a progressive narrowing of the unfolding transition.

**Figure 3:**
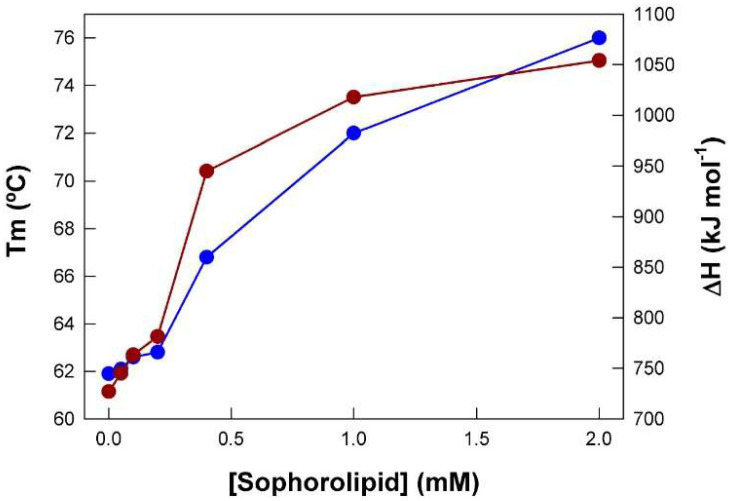
Tm (blue symbols) and ΔH (red symbols) obtained from the thermograms shown in Figure 2 as a function of SL concentration. BSA concentration was 0.1 mM. Data correspond to one representative experiment.

With the Tm and ΔH data obtained from Figure 2, a plot of their dependence on SL concentration was constructed (Figure 3). The thermal unfolding of pure BSA displayed a Tm value of 61.9 °C and a ΔH value of 727 kJ mol^−1^. Concentrations of SL of up to 0.2 mM had a very low effect but upon exceeding this value, there was a steep increase in these two magnitudes, reaching maximum values of ca. 76 °C and 1054 kJ mol^−1^, respectively. Clearly, the shape of the curves suggests a cooperative interaction between SL and BSA.

### 2.2. SL-Induced Structural Changes in BSA

**Figure 4:**
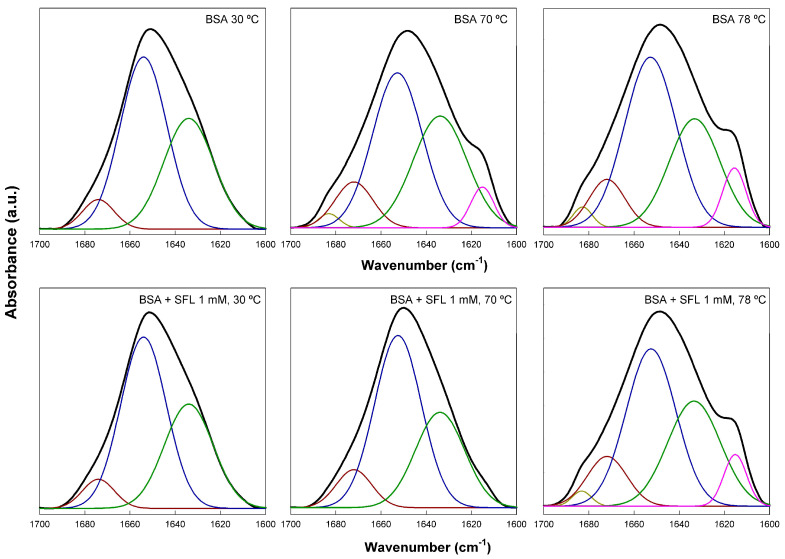
The FTIR amide I’ band of native BSA at various temperatures in the absence and presence of SL. BSA concentration was 0.2 mM and SL concentration was 1 mM. Measurements were carried out in a D_2_O buffer at pD 7.4. Colored lines correspond to the component bands obtained after band fitting, as explained in the text.

By analyzing the amide I’ band in the FTIR spectra of BSA, we could investigate structural changes within the protein after it interacted with SL. The amide I’ band is a complex infrared absorption composed of several overlapping signals (Figure 4). We used derivation and self-deconvolution to pinpoint individual component bands at approximately 1683, 1674, 1654, 1634, and 1615 cm^−1^. These observations corroborated findings from previous studies [22,23,24,25,26]. Native fully hydrated BSA typically contains about 68% α-helix and 31% unordered structure (extended chains), the proportion of β structure being very low. It is worth noting that these data can fluctuate significantly based on physicochemical parameters, concentration, and other variables [24,27,28]. For this study, we largely adopted the band assignments previously established by Sánchez and co-workers [22]: 1683 and 1614 cm^−1^ for unordered chains (which may also indicate intermolecular aggregation), 1654 and 1674 cm^−1^ for α-helices [26], and 1642 and 1634 cm^−1^ for unordered structures, mainly connections between helices. This assignment was carefully determined through a comprehensive review of the existing literature.

Figure 4 illustrates how SL affects the secondary structure of fully hydrated BSA, based on fitting the amide I’ band. We took measurements every 4 °C from 30 to 78 °C, covering temperatures significantly below and above BSA’s denaturation point. For simplicity, Figure 4 only displays spectra at 30, 70, and 78 °C. At 30 °C, native BSA’s spectrum revealed 61.8% helical structure (1674 and 1654 cm^−1^ bands) and 38.2% extended or unordered chains (1634 cm^−1^ band), with virtually no β-structure. These findings align well with previously published data [22,24,27,28]. When the protein was heated to 70 °C, we observed a shift in its secondary structure; aggregated β structures (7.4%), indicated by shoulders at 1684 and 1615 cm^−1^, began to appear. Concurrently, the α-helix content significantly dropped to 46.2%. Further increasing the temperature to 78 °C resulted in similar structural changes, with a minor additional increase in the 1615 cm^−1^ band. These transformations are attributed to protein unfolding, a well-characterized phenomenon [29]. Adding 1 mM SL to native BSA at 30 °C had little impact on the protein’s structure, which remained 63.3% α-helical and 36.7% unordered. However, SL’s protective effect became very clear at 70 °C; the spectrum closely resembled that of pure BSA at 30 °C, showing 65.5% helical and 34.5% unordered structures. This data unequivocally demonstrates the biosurfactant’s ability to protect BSA from thermal unfolding. This protection vanished when samples were heated to 78 °C, as evidenced by the reappearance of the 1684 and 1615 cm^−1^ bands, indicating that protein unfolding had occurred. Our FTIR data thus showed that the addition of acidic SL biosurfactant to BSA had a protective effect against thermal unfolding (denaturation), not causing significant effects on the protein structure. Various cationic, anionic, and non-ionic surfactants, including SDS and Triton X-100, have been shown to cause considerable changes in the helical content of BSA [19,27,30], indicative of a denaturation effect. The protective interaction of SL with BSA opens a wide field of possible practical applications of this biosurfactant.

**Figure 5:**
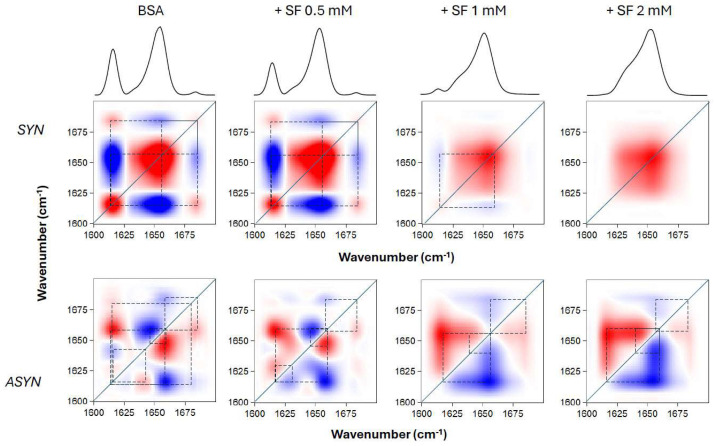
Two-dimensional correlation analysis of the FTIR amide I’ band of BSA upon thermal unfolding, in the absence and presence of SL. BSA concentration was 0.2 mM, and SL was added at various concentrations, as indicated, and incubated. The synchronous (SYN) and asynchronous (ASYN) plots were constructed from the spectra taken at temperatures between 30 and 78 °C, at 4 °C intervals. Red and blue spots correspond to positive and negative peaks, respectively. Dashed lines are depicted to indicate the connections between the various cross peaks.

To understand how the different temperature-induced conformational changes in BSA are related, we performed a 2D correlation analysis of the FTIR spectra. This technique helped us achieve two main goals: first, to identify which specific structural features (represented by component bands of the amide I’ band) correlated with temperature changes (using synchronous spectra) and second, to determine the sequential order of these events (using asynchronous spectra). Figure 5 displays the 2D-IR maps of the amide I’ region. Both pure BSA and BSA with varying SL concentrations showed intense autopeaks at 1615 and 1654 cm^−1^ in their synchronous spectra. This indicates that the most significant secondary structural changes in the protein occurred at these frequencies. The negative cross peaks observed for these two frequencies mean they were not synchronized; specifically, the decrease in helical content correlated with an increase in aggregated structures as the temperature rose. In contrast, positive cross peaks would indicate synchronized changes, meaning they either increased or decreased simultaneously with changes in the external variable. Interestingly, as SL concentration increased to 1 and 2 mM (while BSA concentration remained constant), the intensity of some bands decreased, becoming almost undetectable, due to the conversion of one secondary structure into another, as mentioned above. A clear observation from the asynchronous maps was that the presence of SL fundamentally changed the sequence of structural events in BSA during heating. Without SL, native BSA’s most intense cross peaks were at 1654, 1642, and 1615 cm^−1^. The signs of these cross peaks revealed a specific sequence of events upon heating: 1654 → 1642 → 1615 cm^−1^. This data clearly showed a distinct order during the protein’s thermal unfolding; changes in the helical regions occurred earlier than those in the unordered and aggregated β-structures. This pattern is consistent with previous findings for BSA, even considering slight differences in experimental setup [25]. In contrast, when SL was introduced, the 1615 cm^−1^ band showed changes distinctly prior to the 1654 cm^−1^ band, signifying that β-aggregation preceded the unfolding of the helical domains. This finding supports the idea that SL’s binding to BSA’s helical regions conferred protection against thermal denaturation.

### 2.3. Computational Docking

**Figure 6:**
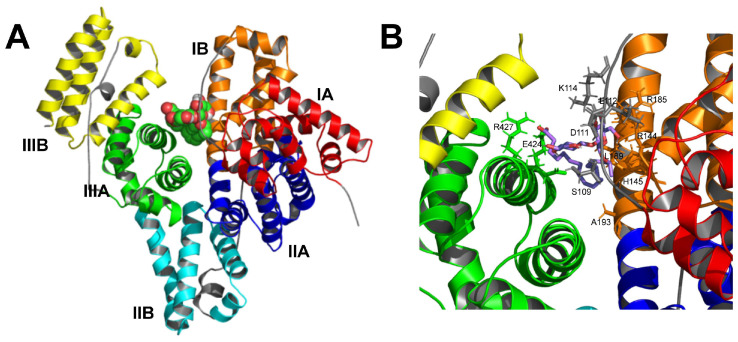
Docking of SL to BSA. (**A**) 3D representation of the whole protein showing the secondary structure as α-helices in different colors: subdomain IA red, IB orange, IIA blue, IIB cyan, IIIA green, and IIIB yellow. SL is depicted in balls, carbon atoms in green, and oxygen atoms in red. (**B**) Close-up of the ligand binding pocket. The carbon skeleton of SL is shown in violet sticks.

Docking of SL to BSA is shown in Figure 6. The selected docking position is the one with the lowest binding free energy found, i.e., −1.58 kJ·mol^−1^, which corresponds to a dissociation equilibrium constant, K_d_, of 14.5 μM. This is a very low K_d_, indicating the high affinity of SL to bind BSA. The binding pocket appears to be located approximately in the center of the protein structure (Figure 6A), where multiple SL-BSA interactions can be observed (Figure 6B), resulting in a low K_d_ value.

The possible ligand–protein interactions observed are summarized in Table 1. The α helices or binding loops containing the amino acid residues are shown, along with their distance (Å) to the SL molecule. SL could bind to different parts of the BSA structure, connecting α-helices 8, 10, and 23, and loops 6–7 and 8–9, via hydrophobic interactions, salt bridges, and hydrogen bonds (Table 1).

**Figure 7:**
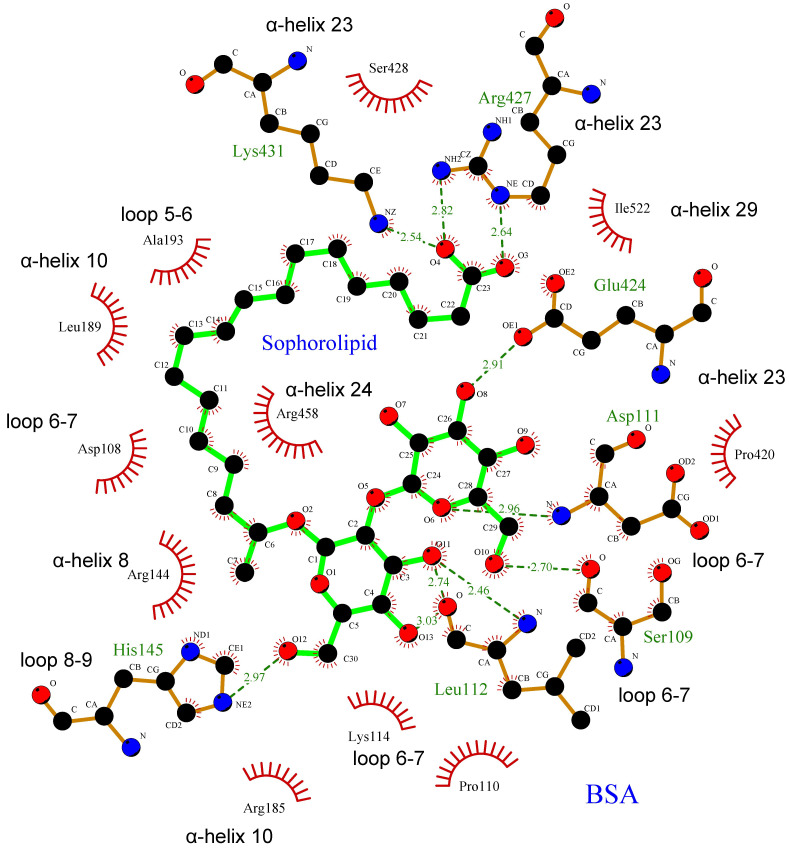
Two-dimensional docked conformation of SL to BSA. Oxygen, carbon, and nitrogen atoms are colored red, black, and blue, respectively. The green solid lines stand for SL, while brown solid lines stand for amino acid residues belonging to BSA. The dotted lines show the hydrogen bonds including the bond length (Å). The red solid wire comb patterns refer to hydrophobic contacts.

All these interactions can be better observed in the two-dimensional representation in Figure 7. This particular docking position would allow the SL molecule to stabilize the three-dimensional structure of the protein by embracing different regions of the protein from the center of the BSA structure. This result would be in good agreement with the experimental results shown above, in which the presence of SL protects the protein structure against the structural disorganization promoted by the increase in temperature.

### 2.4. Final Considerations on SL-BSA Interaction

The BSA structure has a distinctive heart-shaped structure, made up of three helical domains (I, II, and III) that are similar to each other [31,32,33]. Each of these domains is further divided into two subdomains, labeled A and B, a structural arrangement quite like that of Human Serum Albumin [34].

The interaction regions found in BSA likely correspond mainly to subdomains IB and IIIA (Figure 6A and Table 1), in which hydrophobic interactions and hydrogen bonds were found in subdomain IB, and salt bridges in subdomains IB and IIIA. On the other hand, protein-ligand interactions are reported to occur primarily within the IIA and IIIA subdomains, according to existing research indicating that molecular probes or drugs typically bind at these IIA or IIIA subdomains [32,35,36]. However, the SL binding site lies between subdomains IB and IIIA, probably due to its chemical properties as a biosurfactant glycolipid that confers amphipathic character by containing a carboxyl group and two glycosyl residues as a polar headgroup, and a hydrocarbon side chain. The carboxyl group attaches Arg-427 and Lys-431 in α-helix 23 within subdomain IIIA. Simultaneously, the hydrocarbon side chain engages in hydrophobic interactions with subdomain IB, while the glycosyl residues bind at loop 6–7 in domain I (Table 1 and Figure 7). This unique arrangement not only perfectly suits the amphiphilic SL molecule but also corroborates our experimental observations.

## 3. Materials and Methods

### 3.1. Materials

The non-acetylated 17-L-[(2-O-D-glucopyranosyl-D-glucopyranosyl)oxy]-*cis*-9-octadecenoic acid (acidic SL) was from CarboSynth (Berkshire, UK). According to the certificate of analysis given by the supplier, the purity of non-acetylated acidic SL was 90.4%, as determined by HPLC; the dry matter (IR balance at 105 °C) was 98.7%; and free fatty acid content was <0.001%. Non-acetylated SL structural characterization was carried out as published before [37]. Bovine serum albumin (BSA) heat shock fraction, protein, fatty acid, and globulin free were from Merck (Rahway, NJ, USA) (min. 99%). All the other reagents used were of the highest purity available. Stock solutions of SL were prepared in chloroform/methanol (1:1) and stored at −20 °C.

### 3.2. High-Sensitivity Differential Scanning Calorimetry

The thermal denaturation of BSA was monitored by differential scanning calorimetry (DSC) using a VP-DSC high-sensitivity differential scanning calorimeter from MicroCal (Northampton, MA, USA), provided with a self-contained pressurizing system. Thermograms were recorded between 30 and 90 °C at a scan rate of 30 °C h^−1^. It is known that the transition temperature shows some dependence upon scanning rate, particularly in the case of asymmetric endotherms. However, this effect did not affect our experiments since, on the one hand, the endotherms of pure BSA were symmetrical and, on the other hand, the same scan rate was used for pure BSA and BSA in the presence of SL, to make proper comparisons. Furthermore, the scan rate used here has been widely used for these types of studies with BSA and other proteins. The BSA concentration was 0.1 mM (MW 66,300) unless otherwise is indicated. The calorimetric data were analyzed using Origin 7.0 software provided with the equipment, to obtain ΔH and T_m_ values. The curve fitting procedure carried out with some thermograms was performed with Origin software using a Gaussian function.

### 3.3. Fourier Transform Infrared Spectroscopy

The samples for the infrared measurements were prepared essentially as described above in a D_2_O buffer containing 100 mM NaCl and 100 mM phosphate pD 7.4 (pH 7.0). Experiments were performed in triplicate. The BSA concentration was 0.2 mM. The use of Hepes buffer was avoided for these measurements because it presented infrared bands that could interfere with those of the protein. In any case, the use of phosphate buffer did not have any influence on BSA structure and its interaction with SL. An aliquot of the sample (approximately 40 μL) was placed between two CaF_2_ windows using 50 μm Teflon spacers, and the set was mounted in a thermostated Symta cell holder. Infrared spectra were acquired in a Nicolet 6700 Fourier transform infrared spectrometer (FTIR) (Madison, WI, USA). Each spectrum was obtained by collecting 256 interferograms with a nominal resolution of 2 cm^−1^. The equipment was continuously purged with dry air to minimize the contribution peaks of atmospheric water vapor. The sample holder was thermostated using a Peltier device (Proteus system from Nicolet, Green Bay, WI, USA). Spectra were collected at 4 °C intervals, allowing 5 min equilibration between temperatures. The D_2_O buffer spectra taken at the same temperatures were subtracted interactively using either Omnic 8.0 or Grams 7.02 (Galactic Industries, Salem, NH, USA) software. Derivation and Fourier self-deconvolution were applied in order to resolve the component bands of the amide I′ region of the spectrum [38,39]. The secondary structure of the protein was quantified by curve-fitting analysis of the components of the amide I′ band using Grams 7.02 software. During the fitting procedure, the maxima of the bands, determined from deconvolution as explained above, were allowed to move ± 2 cm^−1^.

### 3.4. Computational Docking

Molecular docking of sophorolipid was carried out on BSA. The chemical structure information of sophorolipid was obtained from the PubChem Substance and Compound database [40] through the unique chemical structure identifier CID 11856871. The molecular structure of BSA was taken from the Protein Databank (PDB ID: 4F5S, chain A) at 2.47 Å resolution [41]. Chain A, composed of 583 amino acids and 33 assigned α-helices, was selected from the crystallized dimer. Input protein structure for docking was prepared by adding all hydrogen atoms and removing non-protein molecules such as water and triethylene glycol molecules. Gasteiger atom charges at pH 7 to both ligand and protein, as well as rotatable bonds in the ligand, were assigned by using AutoDockTools4 software [42,43]. The AutoDock 4.2.6 [42] package was employed for docking. The Lamarkian Genetic Algorithm was chosen to search for the best conformers. The number of independent dockings was set to 100, the maximum number of energy evaluations to 25,000,000, the maximum number of generations to 270,000, and the population size to 250. Grid parameter files were built using AutoGrid 4.2.6 [44]. The grid box was selected to include the complete protein structure. The grid box was centered at the center of mass of the protein, with a grid size set to 250 × 185 × 250 grid points (x, y and z), with grid point spacing kept at 0.375 Å. Other AutoDock parameters were used with default values. PyMOL 2.5.0 [45] was employed to edit and inspect the docked conformations. LigPlot 2.3 software was used to calculate protein–ligand interactions and for two-dimensional representations [46].

## 4. Conclusions

SL effectively protects BSA against thermal unfolding or denaturation, enabling the protein to maintain its helical structure at temperatures like 70 °C, unlike pure BSA, which unfolds. Importantly, SL does not cause significant structural changes to native BSA upon initial binding, differentiating it from other surfactants. The presence of SL fundamentally alters the sequence of BSA’s thermal unfolding events; without SL, helical portions change earlier than aggregated structures, but with SL, β-aggregation precedes the unfolding of helical domains. This indicates that SL’s binding to BSA’s helical regions confers protection.

According to docking results, SL binds BSA with high affinity (K_d_ = 14.5 μM), stabilizing its 3D structure against thermal disorganization. The central binding site involves α-helices and loops via hydrophobic interactions, salt bridges, and hydrogen bonds. Uniquely, SL binds between BSA domains IB and IIIA, attributed to its amphipathic nature, differing from typical ligand binding sites.

## Figures and Tables

**Table 1:** Sophorolipid–BSA interactions from docking results.

Interactions	Amino Acids	Domains	Helix/Loop	Distance (Å)
Hydrophobic	Leu-189	IB	10	3.43
	Ala-193	IB	10	3.61
Hydrogen bonds	Ser-109	IA-IB	6–7	1.89
	Asp-111	IA-IB	6–7	2.31
	Leu-112	IA-IB	6–7	1.82
	Arg-144	IB	8	3.21
	His-145	IB	8–9	2.45
	Arg-185	IB	10	2.89
	Glu-424	IIIA	23	2.08
Salt Bridges	Lys-114	IA-IB	6–7	5.00
	His-145	IB	8–9	5.09
	Arg-427	IIIA	23	3.24
	Lys-431	IIIA	23	3.49
Non-bonded Contacts	Asp-108	IA-IB	6–7	2.90
	Pro-110	IA-IB	6–7	2.50
	Pro-420	IIIA	23	2.80
	Ser-428	IIIA	23	2.80
	Arg-458	IIIA	24	3.70
	Ile-522	IIIB	29	2.80

## Data Availability

The original contributions presented in this study are included in the article. Further inquiries can be directed to the corresponding author.

## References

[1] Moutinho L.F., Moura F.R., Silvestre R.C., Romão-Dumaresq A.S. (2021). Microbial biosurfactants: A broad analysis of properties, applications, biosynthesis, and techno-economical assessment of rhamnolipid production. Biotechnol. Prog..

[2] Otzen D.E. (2017). Biosurfactants and surfactants interacting with membranes and proteins: Same but different?. Biochim. Biophys. Acta—Biomembr..

[3] Ribeiro B.G., Guerra J.M.C.C., Sarubbo L.A. (2020). Biosurfactants: Production and application prospects in the food industry. Biotechnol. Prog..

[4] Shu Q., Lou H., Wei T., Liu X., Chen Q. (2021). Contributions of glycolipid biosurfactants and glycolipid-modified materials to antimicrobial strategy: A review. Pharmaceutics.

[5] Inès M., Dhouha G. (2015). Glycolipid biosurfactants: Potential related biomedical and biotechnological applications. Carbohydr. Res..

[6] Roelants S., Solaiman D.K.Y., Ashby R.D., Lodens S., Van Renterghem L., Soetaert W., Lisa V.R., Soetaert W. (2019). Production and Applications of Sophorolipids. Biobased Surfactants.

[7] Borsanyiova M., Patil A., Mukherji R., Prabhune A., Bopegamage S. (2016). Biological activity of sophorolipids and their possible use as antiviral agents. Folia Microbiol..

[8] Vasudevan S., Prabhune A.A. (2018). Photophysical studies on curcumin-sophorolipid nanostructures: Applications in quorum quenching and imaging. R. Soc. Open Sci..

[9] Pal S., Chatterjee N., Das A.K., McClements D.J., Dhar P. (2023). Sophorolipids: A comprehensive review on properties and applications. Adv. Colloid Interface Sci..

[10] Miceli R.T., Corr D.T., Barroso M., Dogra N., Gross R.A., Rebecca Miceli T., David Corr T., Margarida Barroso M., Dogra N., Richard Gross A. (2022). Sophorolipids: Anti-cancer activities and mechanisms. Bioorganic Med. Chem..

[11] Nguyen T.T.L., Edelen A., Neighbors B., Sabatini D.A. (2010). Biocompatible lecithin-based microemulsions with rhamnolipid and sophorolipid biosurfactants: Formulation and potential applications. J. Colloid Interface Sci..

[12] Kanwar R., Gradzielski M., Prevost S., Appavou M.S., Mehta S.K. (2019). Experimental validation of biocompatible nanostructured lipid carriers of sophorolipid: Optimization, characterization and in-vitro evaluation. Colloids Surf. B Biointerfaces.

[13] Valotteau C., Banat I.M., Mitchell C.A., Lydon H., Marchant R., Babonneau F., Pradier C.M., Baccile N., Humblot V. (2017). Antibacterial properties of sophorolipid-modified gold surfaces against Gram positive and Gram negative pathogens. Colloids Surf. B Biointerfaces.

[14] Baccile N., Cuvier A.S., Prévost S., Stevens C.V., Delbeke E., Berton J., Soetaert W., Van Bogaert I.N.A., Roelants S. (2016). Self-Assembly Mechanism of pH-Responsive Glycolipids: Micelles, Fibers, Vesicles, and Bilayers. Langmuir.

[15] Morya V.K., Park J.-H., Kim T.J., Jeon S., Kim E.K. (2013). Production and characterization of low molecular weight sophorolipid under fed-batch culture. Bioresour. Technol..

[16] Singh P.K., Bohr S.S.R., Hatzakis N.S. (2020). Direct observation of sophorolipid micelle docking in model membranes and cells by single particle studies reveals optimal fusion conditions. Biomolecules.

[17] Bjerk T.R., Severino P., Jain S., Marques C., Silva A.M., Pashirova T., Souto E.B. (2021). Biosurfactants: Properties and Applications in Drug Delivery, Biotechnology and Ecotoxicology. Bioengineering.

[18] Borzova V.A., Markossian K.A., Chebotareva N.A., Kleymenov S.Y., Poliansky N.B., Muranov K.O., Stein-Margolina V.A., Shubin V.V., Markov D.I., Kurganov B. (2016). Kinetics of Thermal Denaturation and Aggregation of Bovine Serum Albumin. PLoS ONE.

[19] Singh S.K., Kishore N. (2006). Thermodynamic Insights into the Binding of Triton X-100 to Globular Proteins:  A Calorimetric and Spectroscopic Investigation. J. Phys. Chem. B.

[20] Escribá P.V., González-ros J.M., Goñi F.M., Vigh L., Sánchez-magraner L., Fernández A.M., Busquets X., Horváth I., Barceló-coblijn G. (2008). Membranes: A meeting point for lipids, proteins and therapies. J. Cell. Mol. Med..

[21] Zaragoza A., Teruel J.A., Aranda F.J., Marqués A., Espuny M.J., Manresa Á., Ortiz A. (2012). Interaction of a Rhodococcus sp. Trehalose Lipid Biosurfactant with Model Proteins: Thermodynamic and Structural Changes. Langmuir.

[22] Sánchez M., Aranda F.J., Espuny M.J., Marqués A., Teruel J.A., Manresa Á., Ortiz A. (2008). Thermodynamic and structural changes associated with the interaction of a dirhamnolipid biosurfactant with bovine serum albumin. Langmuir.

[23] Le Gal J.M., Manfait M. (1990). Conformational changes of human serum albumin in vivo induced by free fatty acids as studied by Fourier transform infrared spectroscopy. Biochim. Biophys. Acta—Protein Struct. Mol. Enzymol..

[24] Murayama K., Tomida M. (2004). Heat-induced secondary structure and conformation change of bovine serum albumin investigated by Fourier transform infrared spectroscopy. Biochemistry.

[25] Zhang J., Yan Y.-B. (2005). Bin Probing conformational changes of proteins by quantitative second-derivative infrared spectroscopy. Anal. Biochem..

[26] Jackson M., Mantsch H.H. (1995). The Use and Misuse of FTIR Spectroscopy in the determination of protein structure. Crit. Rev. Biochem. Mol. Biol..

[27] Takeda K., Shigeta M., Aoki K. (1987). Secondary structures of bovine serum albumin in anionic and cationic surfactant solutions. J. Colloid Interface Sci..

[28] Wetzel R., Becker M., Behlke J., Billwitz H., Böhm S., Ebert B., Hamann H., Krumbiegel J., Lassmann G. (1980). Temperature behaviour of human serum albumin. Eur. J. Biochem..

[29] Kuhar N., Umapathy S. (2020). Probing the Stepwise Unfolding of Bovine Serum Albumin Using 2D Correlation Raman Spectroscopic Analysis. Anal. Chem..

[30] Deep S., Ahluwalia J.C. (2001). Interaction of bovine serum albumin with anionic surfactants. Phys. Chem. Chem. Phys..

[31] Majorek K.A., Porebski P.J., Dayal A., Zimmerman M.D., Jablonska K., Stewart A.J., Chruszcz M., Minor W. (2012). Structural and immunologic characterization of bovine, horse, and rabbit serum albumins. Mol. Immunol..

[32] Carter D.C., Ho J.X. (1994). Structure of Serum Albumin. Adv. Protein Chem..

[33] Sudlow G., Birkett D.J., Wade D.N. (1975). The Characterization of Two Specific Drug Binding Sites on Human Serum Albumin. Mol. Pharmacol..

[34] Sugio S., Kashima A., Mochizuki S., Noda M., Kobayashi K. (1999). Crystal structure of human serum albumin at 2.5 Å resolution. Protein Eng..

[35] Amiri M., Jankeje K., Albani J.R. (2010). Characterization of human serum albumin forms with pH. Fluorescence lifetime studies. J. Pharm. Biomed. Anal..

[36] Zhang Y., Li Y., Dong L., Li J., He W., Chen X., Hu Z. (2008). Investigation of the interaction between naringin and human serum albumin. J. Mol. Struct..

[37] Franco Marcelino P.R., Ortiz J., da Silva S.S., Ortiz A. (2021). Interaction of an acidic sophorolipid biosurfactant with phosphatidylcholine model membranes. Colloids Surfaces B Biointerfaces.

[38] Kaupplnen J.K., Moffatt D.J., Mantsch H.H., Cameron D.G. (1981). Fourier Transforms in the Computation of Self-Deconvoluted and First-Order Derivative Spectra of Overlapped Band Contours. Anal. Chem..

[39] Cameron D.G., Moffatt D.J. (1984). Deconvolution, Derivation, and Smoothing of Spectra Using Fourier Transforms. J. Test. Eval..

[40] Kim S., Thiessen P.A., Bolton E.E., Chen J., Fu G., Gindulyte A., Han L., He J., He S., Shoemaker B.A. (2016). PubChem Substance and Compound databases. Nucleic Acids Res..

[41] Bujacz A. (2012). Structures of bovine, equine and leporine serum albumin. Acta Crystallogr. Sect. D Biol. Crystallogr..

[42] Morris G.M., Ruth H., Lindstrom W., Sanner M.F., Belew R.K., Goodsell D.S., Olson A.J. (2009). AutoDock4 and AutoDockTools4: Automated Docking with Selective Receptor Flexibility. J. Comput. Chem..

[43] Sanner M.F. (2005). A component-based software environment for visualizing large macromolecular assemblies. Structure.

[44] Huey R., Morris G.M., Olson A.J., Goodsell D.S. (2007). A semiempirical free energy force field with charge-based desolvation. J. Comput. Chem..

[45] Schrödinger, Inc. (2015). The PyMOL Molecular Graphics System.

[46] Wallace A.C., Laskowski R.A., Thornton J.M. (1995). Ligplot: A program to generate schematic diagrams of protein-ligand interactions. Protein Eng. Des. Sel..

